# Immune responses in retinal gene therapy: challenges, mechanisms, and future strategies

**DOI:** 10.3389/fimmu.2025.1664968

**Published:** 2025-10-15

**Authors:** Yawei Ma, Yin Shen

**Affiliations:** ^1^ Eye Center, Renmin Hospital of Wuhan University, Wuhan, Hubei, China; ^2^ Frontier Science Center for Immunology and Metabolism, Medical Research Institute, Wuhan University, Wuhan, Hubei, China

**Keywords:** retinal gene therapy, immune responses, inherited retinal diseases, adeno-associated virus (AAV), immunomodulation

## Abstract

Retinal gene therapy has advanced significantly, offering potential treatments for inherited retinal diseases (IRDs) such as retinitis pigmentosa, which previously lacked effective interventions. Central to this progress are adeno-associated virus (AAV)-based delivery systems, which have become the primary platform for ocular gene therapy due to their favorable safety profile, ability to target specific retinal cell types, and long-lasting therapeutic effects. However, accumulating evidence reveals that even “immune-privileged” retinal microenvironments are not exempt from immune challenges, affecting both the safety and efficacy of these therapies. Both innate immune pathways and adaptive responses can induce intraocular inflammation, leading to reduced transgene expression and compromised treatment. Understanding how these immune mechanisms interact with therapeutic outcomes is crucial for developing effective intervention strategies. This review examines evidence from both animal models and human trials to explore how immune activation affects treatment efficacy across various delivery methods and vector designs. We also assess emerging strategies aimed at protecting retinal function while reducing systemic toxicity.

## 1 Introduction

Inherited retinal diseases (IRDs) are a diverse group of genetic disorders that cause progressive retinal degeneration and irreversible vision loss. Affecting an estimated 5 to 10 million people worldwide, with a prevalence of 0.06% to 0.2%, IRDs pose a significant public health challenge (1). While no curative treatments currently exist, gene therapy has emerged as a promising approach to preserving or restoring vision by delivering functional genes to affected retinal cells. Among various gene delivery methods, adeno-associated virus (AAV) vectors have gained prominence due to their ability to efficiently deliver genes, sustain long-term expression, and maintain a strong safety profile with minimal risk of integrating into the host genome (2). However, immune responses to AAV vectors remain a major challenge. Pre-existing neutralizing Antibodies (NAbs), along with innate and adaptive immune activation following vector administration, can reduce transduction efficiency, trigger inflammation, and ultimately affect treatment success.

**Figure 1:**
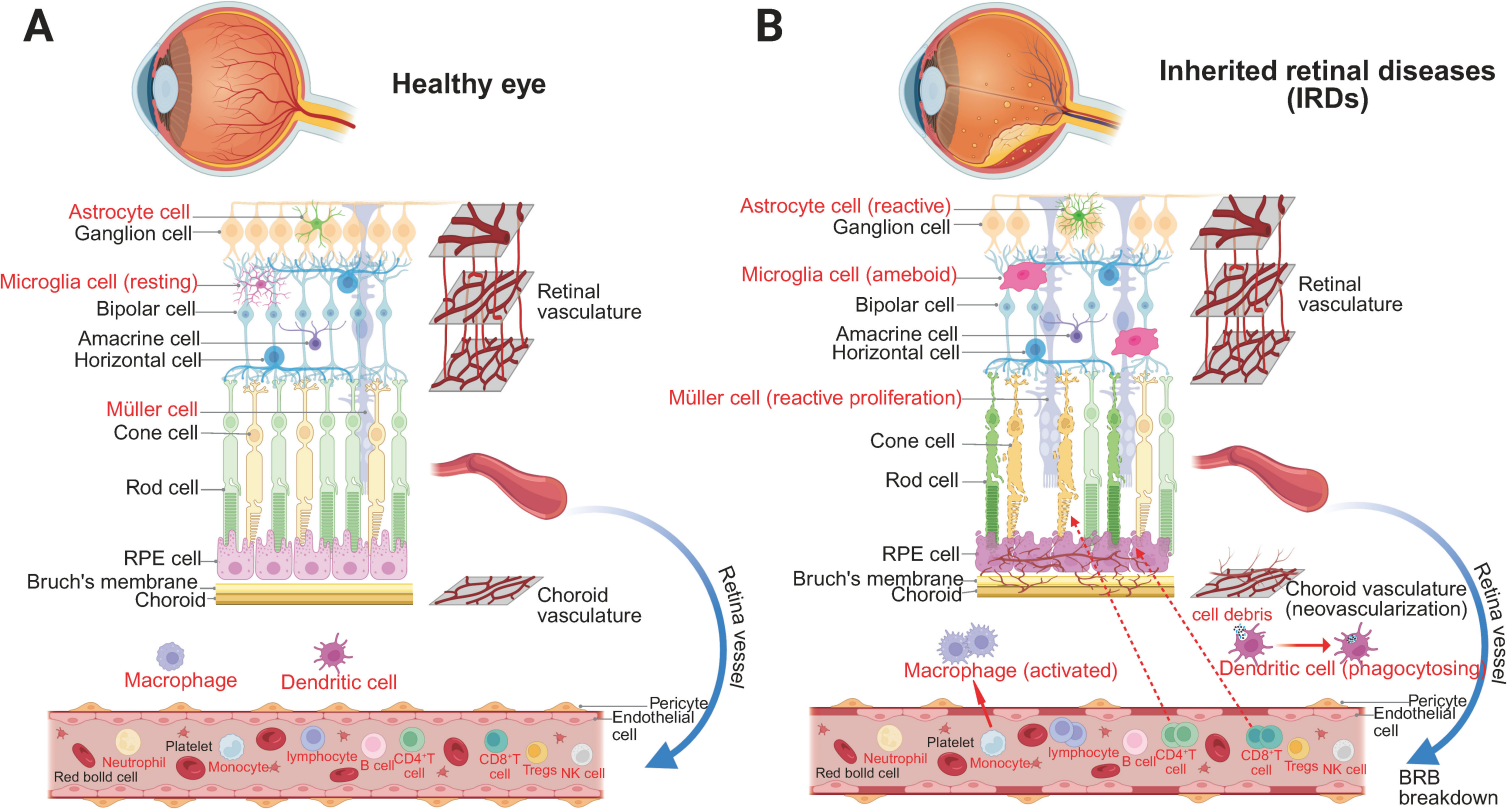
The immune microenvironment of the retina in two different conditions. **(A)** Homeostatic retinal immune microenvironment. Under physiological conditions, the retinal structure is well-preserved and tightly regulated. Astrocytes, resting microglia, and Müller cells maintain homeostasis by supporting the BRB, monitoring neural integrity, and regulating the ionic microenvironment. The BRB effectively restricts the entry of systemic immune cells and inflammatory mediators, ensuring immune quiescence and retinal stability. **(B)** Retinal immune microenvironment in IRDs. In IRDs, progressive degeneration of photoreceptors, bipolar cells, and ganglion cells disrupts retinal architecture, accompanied by RPE dysfunction and gliosis. Astrocytes become reactive, microglia transition to an ameboid morphology, and Müller cells undergo reactive gliosis and proliferation. Breakdown of the BRB, due to pericyte and endothelial cell loss, leads to vascular leakage and pathological neovascularization. Macrophages and dendritic cells actively phagocytose cellular debris, while infiltrating CD4^+^ and CD8^+^ T cells contribute to local immune activation, exacerbating retinal inflammation and degeneration.

**Figure 2:**
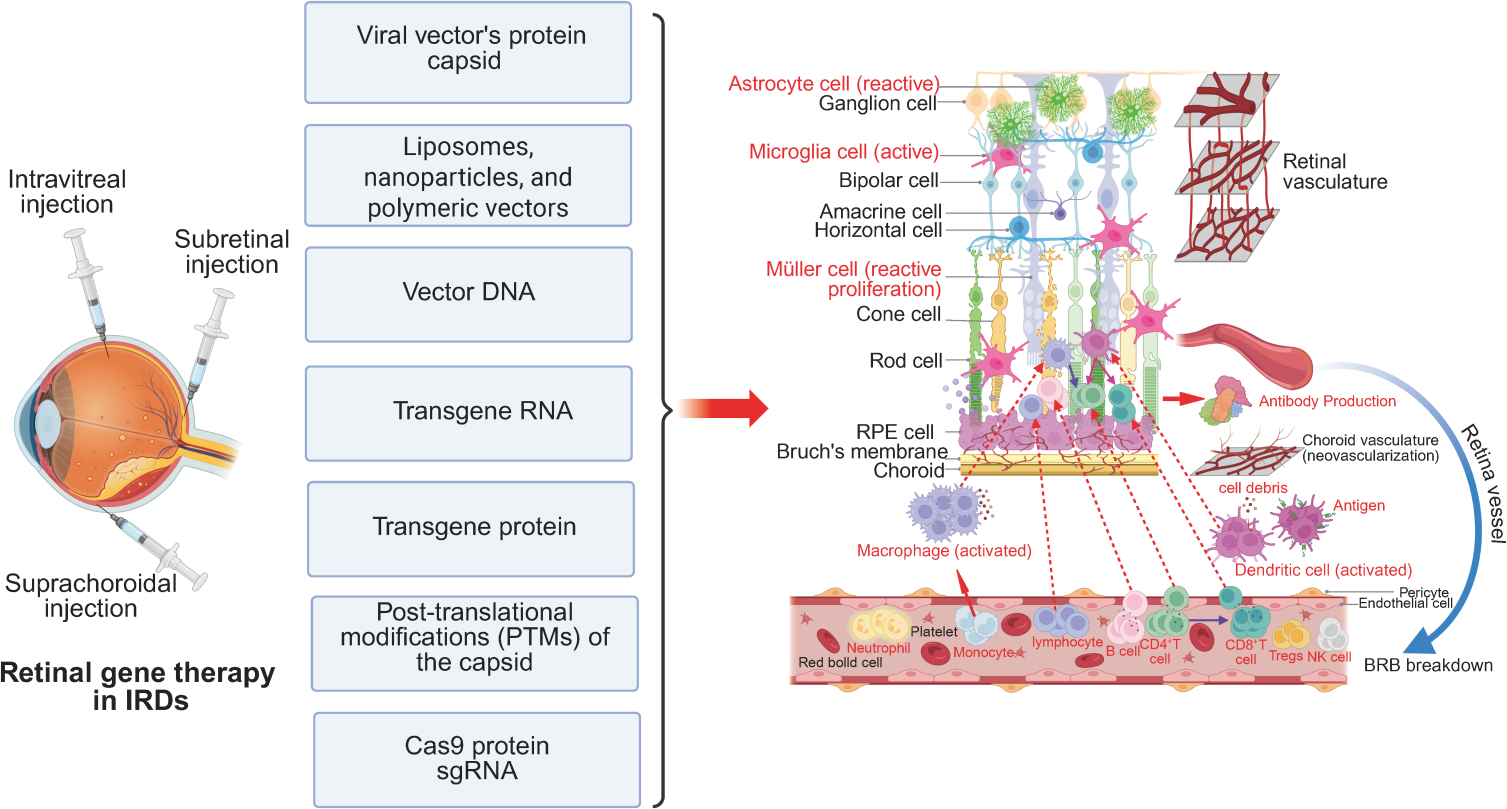
Retinal immune microenvironment in IRDs following gene therapy. Retinal gene therapy is commonly administered via intravitreal, subretinal, or suprachoroidal injection. These delivery methods introduce immunogenic components, including viral capsid proteins, liposomes, nanoparticles, polymeric vectors, vector DNA, transgene RNA, transgene-derived proteins, PTMs of the capsid, and CRISPR-associated proteins such as Cas9 and sgRNA. While therapeutic transgene expression may facilitate partial restoration of retinal function, it can also exacerbate immune activation. Astrocytes, microglia, and Müller cells undergo heightened reactive gliosis and proliferation. Additionally, increased infiltration and activation of peripheral immune cells, including macrophages, dendritic cells, and T lymphocytes, alter the local immune microenvironment, potentially impacting therapeutic efficacy and long-term retinal homeostasis.

Although the retina is traditionally considered an immune-privileged site, IRDs alter its immune microenvironment in ways that may influence vector-mediated immune responses. **Figures 1** and **2** illustrate these changes, highlighting the immune landscape in IRDs and the impact of gene therapy. This review provides a comprehensive analysis of immune responses in retinal gene therapy and explores emerging strategies to overcome these immune related barriers.

To ensure appropriate scope and depth, we conducted a structured search across PubMed, Web of Science Core Collection, and Scopus focusing on IRDs, AAV mediated gene therapy, and immune responses, complemented by targeted screening of ClinicalTrials.gov and backward citation chaining. Queries combined controlled vocabulary and free text terms, including retinal dystrophy and retinal degeneration, IRDs, AAV, gene therapy, NAbs, innate immunity, adaptive immunity, intravitreal delivery, and subretinal delivery, covering January 1990 to June 2025. We included peer reviewed original studies, methods papers, reviews, and clinical trials that directly addressed the topic, and we excluded conference abstracts, duplicates, studies lacking primary data, and studies with low topical relevance.

## 2 Current status of AAV-based retinal gene therapy clinical trials

### 2.1 Ongoing clinical trials

AAV-mediated gene therapy has made significant strides in treating IRDs and age-related retinal degenerative disorders. Currently, most clinical trials are in phases I and II, with fewer advancing to phase III. The ongoing clinical trials, summarized in **Table 1**, use various AAV serotypes (AAV2, AAV5, AAV8, AAV2/4, AAV2/5, AAV2(tYF)), target different genes (e.g., RPE65, REP1, RPGR, sFLT1, VEGF, RS1), and employ diverse delivery methods, including subretinal injection (SRI) and intravitreal injection (IVT). A significant challenge in these trials is the immune response triggered against both the AAV capsid and the transgene product, which can affect the efficacy and safety of the therapy. While the overall incidence of immune-related adverse events is relatively low, most cases are mild. The majority of inflammatory cases involve the entire eye, including both the anterior and posterior segments, while a smaller proportion are limited to the anterior segment. Overall, Immune responses to retinal diseases are variable, depending on the disease type, the patient’s genetic background, and the specific gene targeted.

**Table 1:** Current clinical trials of retinal gene therapy and the associated immune responses.

NCT number	Serotype/vector	Gene target	Dose	Administration	Immune response
NCT02781480	AAV2/5	RPE65	1x10^11^ vg, 3×10^11^ vg, 1×10^12^ vg	SRI	Anterior segment inflammation
NCT00749957	AAV2	RPE65	1.8×10^11^ vg, 6×10^11^ vg	SRI	Inflammation
NCT00999609	AAV2	RPE65	1.5×10^11^ vg	SRI	Anterior segment inflammation
NCT01496040	AAV2/4	RPE65	1.22×10^10^ to 4.8×10^10^ vg	SRI	Inflammation
NCT00643747	AAV2	RPE65	1×10^11^ vg, 1×10^12^ vg	SRI	Anterior & posterior segment inflammation
NCT00481546	AAV2	RPE65	5.96×10^10^ to 1.79×10^11^ vg	SRI	Anterior & posterior segment inflammation
NCT02553135	AAV2	REP1	1×10^11^ vg	SRI	Anterior & posterior segment inflammation
NCT02077361	AAV2	REP1	1×10^11^ vg	SRI	Inflammation
NCT03496012	AAV2	REP1	1×10^10^ vg, 1×10^11^ vg	SRI	Anterior & posterior segment inflammation
NCT03507686	AAV2	REP1	1×10^11^ vg	SRI	Anterior & posterior segment inflammation
NCT02407678	AAV2	REP1	1×10^11^ vg	SRI	Anterior & posterior segment inflammation
NCT03252847	AAV5	RPGR	1×10^11^ vg, 2×10^11^ vg, 4×10^11^ vg	SRI	Anterior & posterior segment inflammation
NCT03116113	AAV8	RPGR	5×10^9^ vg to 5×10^11^ vg	SRI	Anterior segment inflammation
NCT01494805	AAV2	sFLT1	1×10^11^ vg	SRI	Anterior segment inflammation
NCT01024998	AAV2	sFLT1	2×10^8^ vg to 2×10^10^ vg	IVT	Anterior & posterior segment inflammation
NCT03066258	AAV8	VEGF-mAb fragment	3×10^9^ vg to 2.5×10^11^ vg	SRI	Anterior & posterior segment inflammation
NCT02416622	AAV2(tYF)	RS1	1×10^11^ vg, 3×10^11^vg, 6×10^11^ vg	IVT	Anterior & posterior segment inflammation

vg, virus vector genomes; SRI, subretinal injection; IVT, intravitreal injection.

### 2.2 Influence of dose, serotype, administration, and immunosuppression on immune responses in AAV clinical trials

AAV retinal gene therapy has demonstrated promising efficacy across various indications. However, factors such as dose, serotype, and administration route significantly influence both gene expression and the immune response. As the dose of AAV vectors increases, the incidence and severity of immune responses also rise. When the AAV dose exceeds 1×10¹¹ vg/eye, ocular immune responses and severe complications significantly increase (3). In human IVT trials for X-linked retinoschisis (AAV8-RS1), dose-related ocular inflammation was observed, with all subjects at 1×10¹¹–3×10¹¹ vg/eye developing intraocular inflammation in one study (4), whereas another report noted that doses up to 1×10¹¹ vg/eye were generally tolerated but still showed dose related ocular events (5).

Clinical data also indicate serotype dependent differences in immune responses. AAV2, including engineered intravitreal variants such as 2.7m8, has shown more pronounced inflammatory signals in humans. In a diabetic macular edema program employing anti VEGF expression, severe inflammation and ocular hypotony led to early termination, whereas cohorts with neovascular age related macular degeneration exhibited milder steroid responsive inflammation over longer term follow up (6). AAV8 in human XLRS (AAV8-RS1) produced inflammatory events that were generally controllable (5). In patients with X linked retinitis pigmentosa, AAV5 hRKp.RPGR (Botaretigene Sparoparvovec) has predominantly shown manageable inflammation alongside functional signals in phase I and II studies (7).

SRI is the most common route for retinal gene therapy, carrying a lower risk of systemic immune responses than IVT. However, it can still cause localized retinal inflammation, typically mild anterior chamber reactions such as uveitis, and a temporary rise in anti-AAV NAbs. In contrast, IVT elicits a stronger immune response, especially at higher doses. In the NCT01024998 trial, 12 of 19 patients developed anti-AAV2 antibodies, potentially reducing treatment efficacy (8). This highlights the greater immune challenge of IVT and the need for stronger immunosuppressive strategies. Additionally, IVT induces higher NAb levels in serum and ocular fluids, which may impact second-eye treatment success (9).

Additionally, immune management and long-term monitoring are crucial considerations. Most patients exhibit mild immune responses, typically resolved over time either with corticosteroid (7) or even without intervention. Most treatment protocols involve the use of corticosteroids during the first two months following retinal gene therapy. While corticosteroids are effective in controlling inflammation, their long-term use can result in complications such as increased intraocular pressure (IOP), cataracts, and other immune suppression-related issues (10). Therefore, it is crucial to closely monitor patients for these potential side effects to ensure treatment safety. Given the limitations associated with corticosteroid use, future research should focus on developing more targeted and less invasive immunomodulatory strategies.

Many current clinical trials have short follow-up periods, leaving the long-term immune response and durability of treatment effects unclear. Continuous monitoring of immune responses, the persistence of AAV vectors, and overall efficacy is therefore essential to ensure the long-term safety and effectiveness of gene therapy. Some clinical trials have not provided detailed immune response data and treatment effectiveness. Future studies should combine immune response with clinical outcomes, vision improvement and retinal function recovery, to comprehensively evaluate the success of gene therapy. The impact of repeated dosing in the same eye on immune responses is still uncertain and requires further investigation. Additionally, optimizing immunosuppressive protocols, such as adjusting corticosteroid dosing and duration, should be a priority. Exploring the use of low-immunogenic AAV vectors will also be crucial to minimizing the impact of immune responses on the effectiveness of gene therapy.

## 3 Current challenges in retinal gene therapy

### 3.1 Immunogenic component

#### 3.1.1 Viral vector capsid

The variation in the capsid structure of viral vectors, especially AAV vectors, has a significant impact on immune responses. Different serotypes of AAV have unique capsid structures, which determine their ability to target specific tissues by binding to specific receptors on host cell surfaces. These structural variations affect antigenic properties, viral entry mechanisms, targeting specificity, and transduction efficiency. AAV2 is the earliest and most widely used AAV serotype. It enters cells by binding to heparan sulfate, a type of glycosaminoglycan expressed on the surface of host cells, effectively transducing RPE cells (11). AAV5 and AAV8 serotypes are more effective in transducing retinal photoreceptor cells (12).

After AAV vectors enter the body, the host immune system recognizes specific antigenic epitopes on their capsid proteins such as the major structural proteins VP1, VP2, and VP3 of AAV (13). B cells produce specific antibodies (mainly IgG) against these antigens. These antibodies can bind to AAV vectors, forming antigen-antibody immune complexes, which then trigger the activation of the complement system. Complement activation by AAV IgG immune complexes has been demonstrated in human sera, including with AAV9, generating C3a and C5a and highlighting a potential risk of inflammatory injury at high vector doses (14).The deposition of C3b enhances the clearance of immune complexes and may also form the membrane attack complex (MAC), causing direct damage to the AAV vector and the transduced cells (15). In terms of cellular immunity, variations in the AAV capsid can affect the activation of CD4^+^ T cells and CD8^+^ T cells. The activation of these T cells can trigger more severe inflammatory responses and immune-mediated tissue damage. In murine intravitreal studies, administration of AAV2 provokes clinically evident vitritis within about one week and this largely resolves by about one month, whereas subclinical CD45^+^ infiltration, predominantly T cells, persists; prior exposure to AAV accelerates these adaptive responses (16). In particular, the cytotoxic responses mediated by CD8^+^ T cells may directly damage transduced cells, affecting treatment outcomes (17). Evidence from non-ocular clinical studies shows expansion of capsid specific CD8^+^ T cells after AAV exposure and loss of transduced cells in a hemophilia B trial, directly linking capsid antigen presentation to T cell mediated cytotoxic clearance (18).

#### 3.1.2 Vector DNA

The structure and sequence of rAAV genomes critically shape innate sensing and downstream adaptive responses. Across mouse hepatic and pulmonary models, self-complementary genomes expose CpG motifs more efficiently than single stranded genomes and consequently elicit stronger endosomal TLR9 dependent MyD88 signaling, type I interferon (IFN) responses, and capsid specific CD8^+^T cell priming; these effects are markedly blunted in Tlr9 deficient or Myd88 deficient mice (19–21). The GC-rich palindromic inverted terminal repeat (ITR) hairpins, while indispensable for replication and packaging, also harbor CpG motifs recognized by TLR9. Genome engineering strategies, including CpG depletion within the expression cassette and ITRs and the insertion of short TLR9 inhibitory oligomers, reduce inflammatory readouts in mouse liver, muscle, and retina, and after subretinal delivery in pigs. By contrast, in macaques, intravitreal dosing delayed but did not prevent uveitis, underscoring route and species dependent constraints (22, 23).

Beyond TLR9, interspecies differences in cytosolic DNA sensing complicate translation. Murine cells depend mainly on the cGAS-STING pathway, whereas human cells also elicit a strong STING independent response mediated by DNA dependent protein kinase, a kinase that can sense DNA ends and initiate innate signaling (24). Finally, promoter and transgene features, including length, GC content and CpG content, and predicted secondary structure, modulate both expression and engagement of TLR9. CpG depleted cassettes dampen TLR9 dependent cross priming without compromising expression in muscle and liver. CpG depleted ITRs have retained vector activity *in vivo* (20, 25). Consistent with these model data, human whole blood assays indicate that plasmacytoid dendritic cell TLR9 dependent type I IFN responses to AAV9 require pre-existing anti AAV9 antibodies, highlighting the influence of host humoral background on genome sensing (26).

#### 3.1.3 Transgene RNA

Beyond the capsid and vector DNA, vector encoded transcripts are not immunological bystanders. Engagement of pattern recognition receptors (PRRs) and the magnitude of downstream interferon programs are determined by intrinsic RNA features, including length, sequence composition such as GU richness, chemical modifications, and higher order structure. Within endosomes, TLR3 senses long double stranded RNA, as shown in Tlr3 deficient mice and in polyinosinic polycytidylic acid challenge systems, whereas TLR7 and TLR8 detect GU rich single stranded RNA with pronounced species asymmetry. In mice, responses are dominated by TLR7 and TLR8 activity is weak or non-canonical. In humans, TLR8 is strongly responsive to single stranded RNA in primary monocytes, myeloid dendritic cells, and peripheral blood mononuclear cells (27–29).

In the cytosol, RIG I preferentially recognizes short RNAs bearing a5′-triphosphorylate, including double stranded and single stranded species, whereas MDA5 senses long double stranded RNA, and both receptors converge on MAVS. These assignments are supported by studies in knockout mice, human primary cells, and biochemical mapping of structure and length determinants (30, 31). Relevant to AAV, sustained transduction can generate antisense or minus-strand transcripts from the vector template, enabling sense–antisense pairing and dsRNA biogenesis. Across transformed cell lines (HeLa, HEK293, HepG2, Huh7), primary human hepatocytes (about 10–12 donors), and mice bearing human hepatocyte xenografts, dsRNA engages MDA5, signals through MAVS, and induces IFN-β, thereby curtailing transgene expression. siRNA knockdown of MDA5 or MAVS restores expression *in vitro*, directly linking transgene derived double stranded RNA to innate activation in human cells and in humanized mice (32). Collectively, these findings establish transgene RNA quality, not capsid alone, as a tunable driver of AAV immunogenicity and durability, and highlight the requirement to factor specie specific TLR7 and 8 biology into translational inference.

#### 3.1.4 Transgene protein

Transgenic proteins, as the final expression product of gene therapy, may be recognized as antigens by the host immune system, triggering a specific immune response. The strength and nature of this response depend on several factors related to the transgenic protein, including its structure, function, stability, and folding state (33). In particular, the glycosylation patterns and folding quality of the transgenic protein play an important role in its immunogenicity. For example, abnormal glycosylation patterns may cause the transgenic protein to be identified as a foreign antigen, while a protein that is not fully folded may expose atypical epitopes that can be recognized by the immune system. Detection techniques, such as proteomics (34) and ELISA, can be used in laboratory settings to assess the immunogenicity of proteins.

#### 3.1.5 Post-translational modifications of the protein capsid

Viral vector capsids can undergo various PTMs, such as phosphorylation, glycosylation, and ubiquitination (35). These modifications can significantly alter the capsid’s three-dimensional structure, affecting its antigenicity and immunogenicity (36). Different types of PTMs may lead to changes in the immunogenicity of the viral vector in the body, which can impact both the safety and therapeutic effectiveness of gene therapy (37).

### 3.2 Pre-existing immunity against AAV

In AAV mediated gene therapy, pre-existing antibodies against AAV capsid proteins pose a significant challenge. Many individuals have anti-AAV antibodies from previous infections, which reduces the effectiveness of gene therapy by neutralizing viral vectors (38). Because AAV capsid proteins have highly conserved amino acid sequences, NAbs may cross-react with multiple serotypes (39). Vectors containing exons and regulatory sequences from non-human sources can be recognized as foreign by the immune system (40). Furthermore, degradation products of AAV vectors and residual materials from vector production can provoke immune responses (41).

### 3.3 Immune responses within the eye

The eye is considered an immune-privileged site, with mechanisms such as the blood-retina barrier (BRB) and local immunosuppressive factors that protect against excessive immune responses (42, 43). However, this unique immune environment can pose challenges in retinal gene therapy, especially when using AAV vectors. While the eye generally exhibits a tempered immune response, AAV vectors can still trigger both humoral and cellular immune responses, particularly in patients with pre-existing immunity, leading to the neutralization of the vector and reduced therapeutic efficacy. Additionally, local inflammatory responses may cause retinal damage, complicating the control of immune reactions and affecting the long-term success of gene therapy.

#### 3.3.1 Innate immune response

The innate immune system is the first line of defense against foreign pathogens, and in gene therapy, both viral vectors and gene editing tools like CRISPR/Cas9 and RNA interference (RNAi) can activate innate immune responses. Components of viral vectors, such as the protein capsid (44), vector DNA/RNA sequences (45), Cas proteins, associated small guide RNA sequences (sgRNA), and non-specific RNA degradation products in RNAi technology can be recognized by the host’s PRRs, triggering innate immune responses (46). PRRs are expressed on the cell membrane and in the cytoplasm of various cells, including innate immune cells (e.g., macrophages, monocytes, neutrophils, natural killer cells, and dendritic cells) (47), adaptive immune cells (such as T cells and B cells), and non-immune cells (such as epithelial cells, endothelial cells, and fibroblasts) (48). In the retina, PRRs are predominantly found in retinal pigment epithelial cells (RPE cells) (49), microglial cells (50), retinal ganglion cells (RGCs) (51), retinal astrocytes (52), and photoreceptor cells (53).

Immune responses triggered by AAV vectors involve multiple PRR pathways. TLR2 recognizes the protein capsid of AAV, activating the NF-κB pathway and inducing the production of inflammatory cytokines such as TNF-α and IL-1β (39). Unmethylated CpG motifs within AAV genomes are detected by TLR9, particularly in mouse models where genetic ablation of Tlr9 or Myd88 blunts type-I IFN induction and downstream adaptive immunity (19). In the cytoplasm, RIG-I and MDA5 detect double-stranded RNA produced after AAV infection, which activates type I IFN and NF-κB signaling pathways (32). Additionally, the NLRP3 inflammasome responds to AAV-induced cellular stress and damage by activating caspase-1, leading to the release of IL-1β and IL-18. AIM2 detects double-stranded DNA in the cytoplasm, triggering inflammasome activation (54).

**Figure 3:**
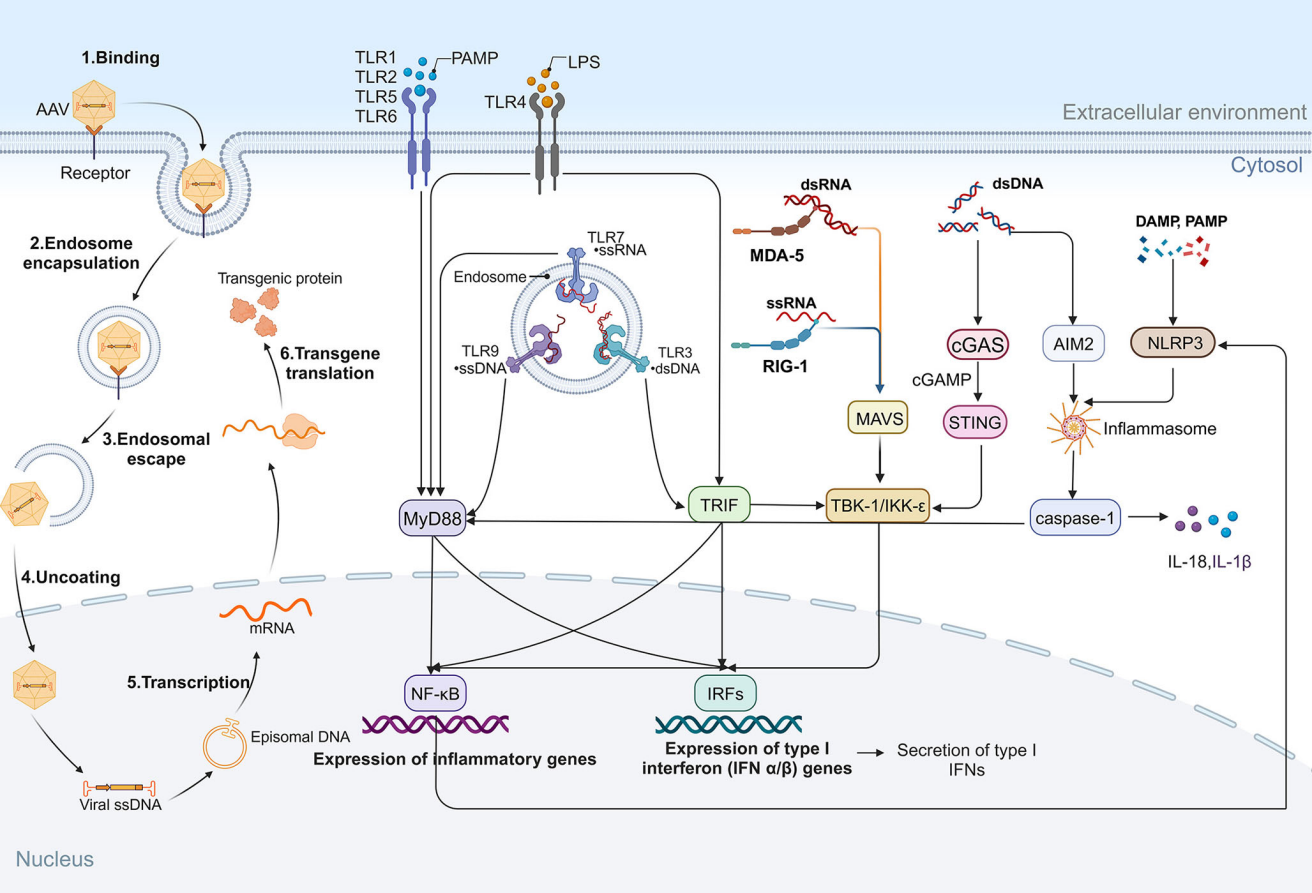
AAV transduction and innate immune responses in retinal gene therapy. Following administration, AAV transduction occurs through sequential steps, including cell surface binding, endocytosis, endosomal escape, capsid uncoating, transcription, and transgene translation. During this process, PRRs detect pathogen-associated molecular patterns (PAMPs) and damage-associated molecular patterns (DAMPs), initiating innate immune responses. TLRs play a key role in immune sensing: TLR1, TLR2, TLR5, and TLR6 recognize PAMPs and activate MyD88-dependent pathways, leading to NF-κB activation and pro-inflammatory gene expression. TLR4, upon recognizing LPS, signals through MyD88 and TRIF, triggering NF-κB and IRFs, resulting in the secretion of inflammatory cytokines and type I IFNs. Additionally, cytoplasmic RNA sensors contribute to immune activation: MDA5 binds dsRNA, while RIG-I binds ssRNA, both of which activate MAVS and TBK1/IKKϵ, promoting NF-κB and IRF signaling. Cytosolic DNA, via the cGAS/STING pathway, activates TBK1/IKKϵ, while also stimulating the AIM2 inflammasome and caspase-1, further amplifying inflammatory cascades. NLRP3 inflammasome activation, triggered by cytoplasmic DAMPs and PAMPs, reinforces inflammatory responses.

Moreover, cGAS recognizes AAV genomic DNA and produces cGAMP, which activates STING. Upon activation, STING induces the production of type I IFNs and inflammatory cytokines. Mouse retina shows cGAS–STING activation *in vivo* (e.g., diabetic retinopathy), supporting the pathway’s relevance to ocular inflammation and its potential contribution to innate responses triggered by vector (55, 56). **Figure 3** shows the AAV transduction process and the innate immune responses triggered in retinal gene therapy. These molecular pathways together form a complex immune response network triggered by various gene therapy methods.

#### 3.3.2 Adaptive immune response

The adaptive immune system (T cells and B cells) is gradually activated within days to weeks after gene therapy, potentially leading to vector clearance, loss of gene expression, and even cytotoxic reactions. In retinal gene therapy, adaptive immune responses mediated by T cells are key to treatment efficacy. CD4^+^ helper T cells activate and regulate immune responses through cytokine secretion, while CD8^+^ cytotoxic T cells directly kill infected cells. Both T cell types are essential for recognizing AAV vector components and transgene products (57).

Antigen-presenting cells (APCs), such as dendritic cells and macrophages, process and present antigenic peptide fragments from viral vectors or transgenes to T cells, triggering T cell activation. After activation, CD8^+^ cytotoxic T lymphocytes (CTLs) proliferate and differentiate, releasing perforins and granzymes to induce apoptosis and kill infected cells (58). During antigen presentation, CD4^+^ T cells recognize viral vector or transgene antigens through MHC II molecules. Upon activation, CD4^+^ T cells differentiate into various subsets, including T helper 1 (Th1), Th2, Th17, and T regulatory cells (Tregs). Each subset plays a distinct role in the immune response (59). T_H_1 cells secrete IFN-γ, which promotes CTL activation and enhances macrophage activity, driving cell-mediated immunity (60). In contrast, Th2 cells produce interleukins such as IL-4, IL-5, and IL-13, which are key for promoting B cell-mediated antibody production and humoral immunity (61). Th17 cells secrete IL-17 and IL-22, involved in inflammatory responses and tissue remodeling processes (62). Tregs secrete immunosuppressive cytokines (IL-10 and TGF-β) to suppress excessive immune responses, prevent uncontrollable inflammation, and maintain immune homeostasis (63).

**Figure 4:**
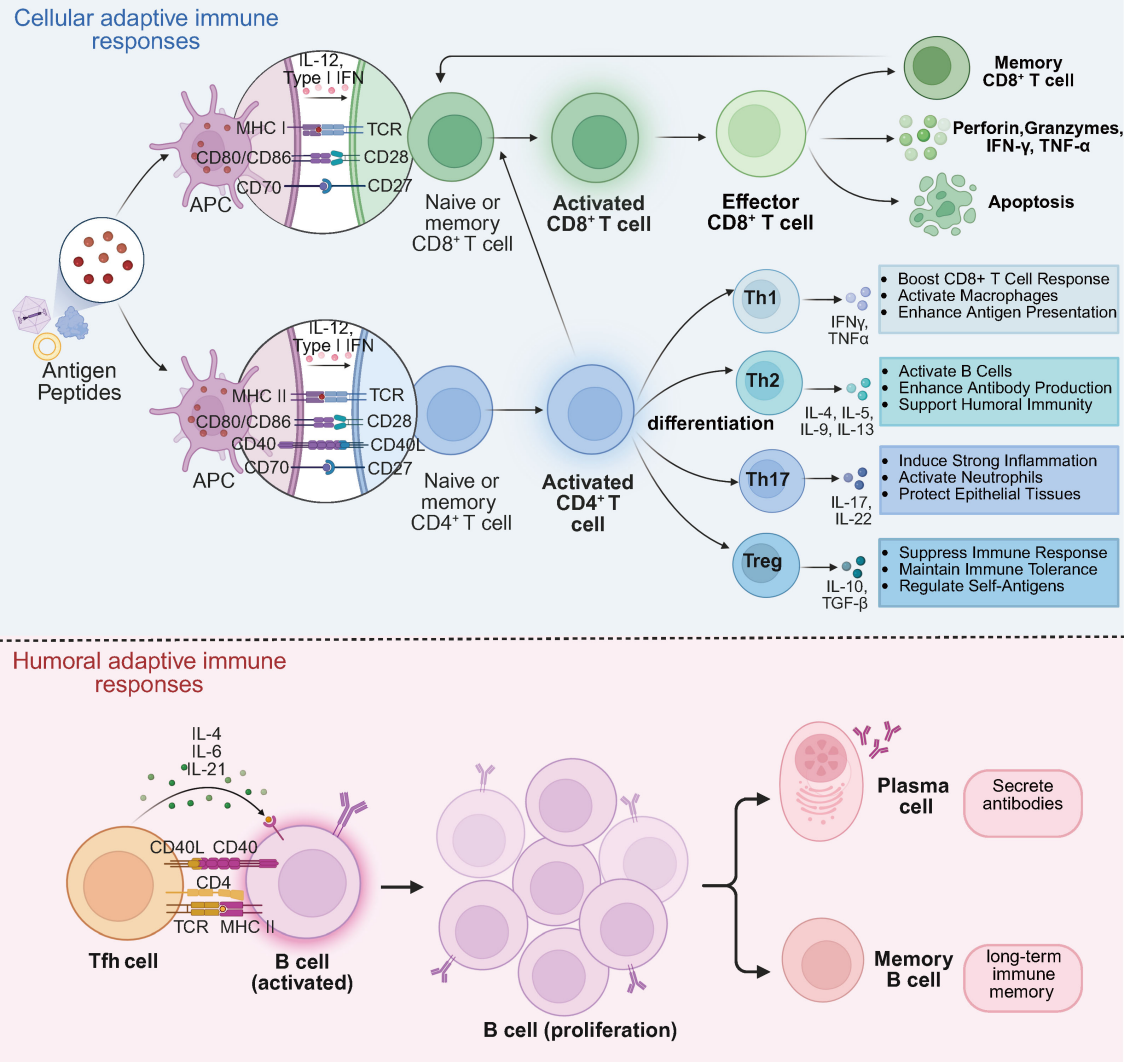
Cellular and humoral adaptive immune responses in retinal gene therapy. Cellular adaptive immune responses. Antigenic peptides derived from gene therapy are presented to naïve and memory CD8^+^ and CD4^+^ T cells via MHC molecules. Naïve and memory CD8^+^ T cells differentiate into activated CD8^+^ T cells, which further develop into effector CD8^+^ T cells. Effector CD8^+^ T cells mediate cytotoxicity by secreting perforin, granzymes, IFN-γ, and TNF-α, leading to apoptosis of target cells. Upon antigen re-exposure, memory CD8^+^ T cells rapidly differentiate into effector or memory subsets, enabling a rapid immune response. Naïve and memory CD4^+^ T cells, upon antigen recognition, differentiate into activated CD4^+^ T cells, which further polarize into Th1, Th2, Th17, and Treg subsets, each secreting distinct cytokines to modulate immune responses. Additionally, activated CD4^+^ T cells enhance CD8^+^ T cell activation, reinforcing cellular immunity. Humoral adaptive immune responses. Tfh cells promote B cell activation, leading to the differentiation of B cells into plasma cells and memory B cells. Plasma cells secrete antigen-specific antibodies, contributing to long-term immune protection, while memory B cells provide rapid recall responses upon re-exposure to the same antigen.

CD8^+^ T cells identify and eliminate target cells expressing viral vector or transgene antigens via MHC I molecules. This process is essential for clearing infected cells and regulating transgene expression (58). Following antigen clearance, some T cells differentiate into memory T cells. Central memory T cells (TCM) and effector memory T cells (TEM) can rapidly respond to subsequent exposures, enhancing immune protection in future gene therapy treatments. B cells play a pivotal role in humoral immunity by recognizing antigens, producing specific antibodies through plasma cell differentiation, and forming memory B cells for long-term immune memory (64). Antibodies neutralize viruses by blocking binding to host cell receptors or by interfering with viral entry into the host cell membrane. This neutralization diminishes the virus’s ability to infect and reduces the transduction efficiency of viral vectors. Furthermore, antibodies can bind to viral antigens on the surface of infected cells, engaging Fc receptors on effector cells such as NK cells, thereby triggering antibody-dependent cellular cytotoxicity (ADCC). Antibodies can also activate the complement system, initiating a cascade that leads to viral lysis and phagocytosis. **Figure 4** illustrates the cellular and humoral immune responses during retinal gene therapy.

### 3.4 Challenges in redosing

Currently, AAV-mediated gene therapy for ocular diseases in clinical settings typically employs a unilateral injection strategy. This approach is primarily driven by safety considerations and the goal of preserving the patient’s vision to the greatest extent possible. However, following a single intravitreal injection of AAV, transgene expression often declines over time due to local or systemic immune-mediated clearance, vector off-target distribution, or suboptimal initial transduction efficiency, making it challenging to achieve durable therapeutic effects. Therefore, once efficacy is confirmed in one eye, safely and effectively redosing AAV in the other eye or the same eye to sustain transgene expression for bilateral or repeated treatment remains a major challenge in ocular gene therapy.

The success of AAV redosing depends on factors such as administration route, vector dose, dosing interval, and recipient age, all of which significantly influence the host immune response and thus determine the outcome of repeated gene delivery. It has been reported that intravitreal injection of AAV induces a humoral immune response against the capsid, which impairs transgene expression upon re-administration via the same intravitreal route, but does not affect transduction following SRI in the contralateral eye. In contrast, subretinal administration does not elicit a humoral immune response and does not interfere with subsequent AAV delivery, whether intravitreal or subretinal (65). Lindsey Weed and colleagues demonstrated in non-human primates (NHPs) that AAV2-hRPE65v2 can be safely and effectively readministered subretinally in the same eye, even in the presence of NAbs, with good local and systemic tolerability (66).

Strategies to overcome humoral immunity and enable effective AAV redosing are actively being explored, building on insights from ocular studies. For example, the IgG-degrading endopeptidase Imlifidase (IdeS) reduces anti-AAV antibody levels, facilitating efficient liver gene transfer in mice and enhancing transduction in NHPs, even after vector re-administration (67). Alternatively, tolerogenic nano-adjuvants (RICP), combining rapamycin (RAPA) and itaconate (ITA), disrupt the T follicular helper cell (Tfh cell) and germinal center B cell axis via metabolic modulation while inducing Tregs, significantly improving hepatic transgene expression upon redosing (68). B cell–targeted approaches also show promise: Anti-CD19 CAR-T therapy depletes peripheral and tissue-resident B cells and plasma cells, eradicating high-titer AAV8 NAbs and permitting successful transgene expression upon systemic AAV8 re-administration in mice (69). Similarly, plasmapheresis offers a clinically feasible method to lower anti-AAV antibodies, particularly for repeat dosing (70). Capsid engineering further expands redosing options. Recent studies demonstrate that modified AAV9 vectors can cross the blood-brain barrier, enabling efficient brain endothelial transduction in mice and NHPs after multiple doses. Although species-specific differences exist, this approach allows serotype switching to circumvent pre-existing immunity during redosing (71).

### 3.5 Personalized immune management

Immune responses can vary significantly between individuals due to factors such as sex, age (72), genetic differences, personal immune history, lifestyle, diet, environmental exposures, immune system variations, and physiological conditions. Personalized immune management is especially important in gene therapy for inherited retinal diseases. By creating personalized treatment plans based on each patient’s genomic data and immune history, genetic variants related to therapeutic responses can be precisely identified, optimizing treatment strategies. The challenge lies in effectively integrating real-time immune monitoring technologies and making timely adjustments to treatment plans based on monitoring results to enhance safety and efficacy. By integrating genomic data and immune biomarkers, more precise treatment plans can be developed. Additionally, a validated IFN-γ ELISpot method facilitate adjustments to treatment based on changes in immune status during clinical trials, thereby improving treatment outcomes and reducing side effects (73).

## 4 Strategies to harness the immune system

### 4.1 Pharmacological and therapeutic interventions

#### 4.1.1 Immunosuppressive drugs

In retinal gene therapy, immunosuppressive drugs play a pivotal role in managing post-treatment inflammation and enhancing safety. Corticosteroids such as dexamethasone and prednisone are commonly used to suppress the release of inflammatory mediators, significantly reducing retinal tissue inflammation. Dexamethasone, well-known for its strong anti-inflammatory effects, inhibits cytokine and chemokine release, thus controlling immune responses to gene therapy (74). Additionally, it induces dendritic cells to exhibit tolerogenic properties, reducing the expression of co-stimulatory molecules and limiting T cell activation (75). Prednisone reduces inflammation by blocking the NF-κB pathway and decreasing inflammatory gene expression (76), offering protection to the retinal microenvironment. Clinical trials also indicate that prednisone helps mitigate chronic inflammation and retinal degeneration over prolonged treatment periods (6). Despite their therapeutic benefits, corticosteroids are not a panacea and present significant limitations. Local administration carries substantial risks, with 20-30% of long-term users developing corticosteroid-induced glaucoma and cataract formation. Systemic administration (oral/intravenous routes) may lead to more extensive complications, including immunosuppression and metabolic disturbances with prolonged use (77).

Beyond steroids, calcineurin inhibitors modulate T cell activation. Cyclosporine A mitigates disease in rat experimental autoimmune uveoretinitis (EAU), supporting a T cell targeted mechanism *in vivo* (78). Tacrolimus (FK506) similarly inhibits calcineurin and reduces ocular inflammation in rabbit and rat EAU after intravitreal delivery, with preservation of retinal structure and electroretinography (ERG) function (79, 80). Although ocular AAV specific tacrolimus data are limited, systemic tacrolimus prolonged rAAV8 and rAAV9 transgene expression in NHPs after skeletal and muscle delivery, indicating translatable T cell control across species and tissues (81, 82). Other agents are being explored. Mycophenolate mofetil inhibits lymphocyte proliferation. However, in mouse liver directed models, it lessens single stranded AAV transduction and may thereby limit overall efficiency (83). These challenges mirror broader findings in AAV gene therapy, where a systematic review of 73 clinical and real-world studies identified immunosuppression optimization as a critical determinant of treatment safety and efficacy (84).

#### 4.1.2 Immune modulation therapy

Immune modulation is essential in controlling immune responses during retinal gene therapy. Immunoglobulin (IVIG), a key immunomodulator, neutralizes pathogenic antibodies and regulates immune functions, significantly reducing immune-mediated adverse reactions. IVIG is commonly used in clinical settings to manage acute immune reactions triggered by AAV vectors by binding to immune molecules, reducing inflammation, and improving treatment outcomes.

Plasmapheresis, though rarely used, can effectively reduce inflammation by removing pathogenic antibodies and immune complexes from the bloodstream, alleviating retinal inflammation (70, 85). In NHPs with pre-existing AAVrh74 immunity, two to three exchanges lowered neutralizing titers and enabled redosing, capsid based immunoadsorption columns also deplete anti-AAV IgG ex vivo and in animal models (70).

Rituximab (anti-CD20) depletes B cells, thereby reducing antibody mediated responses. In mouse and NHP models, B cell depletion, frequently paired with sirolimus, mitigated anti-capsid immunity and permitted AAV redosing. In clinical practice, rituximab is also employed for refractory ocular inflammation (86, 87). mTOR inhibition with rapamycin or sirolimus suppresses T and B cell activity. Phase III trials demonstrated that intravitreal sirolimus improves non-infectious uveitis. In mice and NHPs, rapamycin loaded tolerogenic nanoparticles (ImmTOR) attenuated anti-AAV immunity and enabled vector redosing (88, 89). JAK inhibition similarly reduces inflammatory signaling. Tofacitinib suppresses experimental autoimmune uveitis in mice, and ruxolitinib alleviates endotoxin-induced uveitis in rats, supporting pathway relevance to ocular inflammation even if direct AAV data are preclinical (90, 91). Moreover, immunoenzymes that transiently debulk antibodies are particularly promising in AAV settings. IgG-degrading endopeptidases (IdeS/IdeZ) restore AAV transduction in mice passively immunized with human Ig and permit *in vivo* gene transfer in NHPs in the face of NAbs. IceMG, a dual protease engineered to cleave IgM and IgG, rapidly clears both isotypes in rhesus macaques, reinstates AAV transduction in mice carrying human antisera, and reduces complement activation (67, 92, 93).

#### 4.1.3 Cytokine and signaling pathway modulation

In retinal gene therapy, cytokine modulation plays a key role in managing inflammation. AAV vector injections activate Müller glial cells and microglia, triggering the release of pro-inflammatory cytokines including TNF-α and IL-1β (94). These glia–cytokine dynamics are consistent with broader microglia and Müller biology in mouse retina.

Although not specific to gene therapy, clinical experience in humans with non-infectious uveitis supports targeting these cytokines when intraocular inflammation threatens structure or function. Anti-TNF-α agents (e.g., infliximab) achieve corticosteroid sparing control in refractory uveitis (adult and pediatric cohorts), while anti-IL-6R (tocilizumab) is effective for uveitic cystoid macular edema and in juvenile idiopathic arthritis associated uveitis refractory to anti-TNF therapy. In ophthalmic practice they are generally systemic and used off label to suppress ocular inflammation (95–98).

Targeting the NF-κB and PI3K/Akt signaling pathways can also reduce immune overactivation, providing an additional strategy for immune response modulation. In mouse models of retinal and choroidal inflammation and neovascularization, microglial NF-κB activation is a driver of lesion development. Pharmacologic or genetic dampening of this axis reduces inflammatory infiltration and pathology. In parallel, PI3K/Akt signaling in retinal microglia governs activation and cytokine release, and blocking PI3K (e.g., LY294002) reduces retinal inflammatory programs in the oxygen-induced retinopathy model while dampening BV2 activation. Microglia-focused PI3K regulation (e.g., PIK3IP1) further links this pathway to retinal inflammatory angiogenesis in mouse models. Together, these data support NF-κB and PI3K/Akt as tractable nodes for adjunctive immunomodulation around ocular gene transfer, while acknowledging translation to AAV specific settings remains to be fully defined (99–101).

### 4.2 Vector design and optimization

#### 4.2.1 AAV vector engineering

Modifications to AAV vector capsids are critical in reducing immunogenicity and improving therapeutic efficacy. Glycosylation of the capsid helps mask antigenic epitopes, making the vector less recognizable by the immune system (102). Techniques such as directed evolution and synthetic biology enable the development of AAV variants with lower immunogenicity. Mutations in capsid proteins, sequence recombination, and structural optimization can reduce immune responses while maintaining high transduction efficiency (103, 104). CRISPR/Cas9 technology also facilitates precise modifications to existing AAV vectors, further enhancing their properties (105). Non-viral vectors, such as liposomes and nanoparticles, also hold potential in retinal gene therapy, with modifications in size, surface charge, and composition improving their ability to evade the immune system and deliver genes more efficiently (106, 107). The use of AAV serotypes with low immunogenicity, such as AAV8 and AAV9, is an established strategy to enhance treatment outcomes. These serotypes exhibit high transduction efficiency and reduced immunogenicity in certain tissues (108).

#### 4.2.2 Immune evasion

Polyethylene glycol (PEG) modification of AAV vectors can reduce immune recognition, extending their circulation time in the body (109). Immunomodulatory coatings, such as the regulatory protein CTLA-4, can also be applied to the surface of AAV vectors to reduce immune responses (110). Decoy capsid technology, where immune cells bind to non-therapeutic capsids, can further reduce immune activation against the therapeutic vector (111). Epitope masking, which conceals antigenic epitopes, is another promising approach to reducing immune recognition (112).

## 5 Future directions and clinical implications

### 5.1 Next-generation gene therapy approaches

Emerging non-viral vectors, including lipid nanoparticles (LNPs), polymeric nanoparticles, and mRNA delivery systems, are opening new options for retinal gene therapy across multiple preclinical systems. For example, PEG variant LNPs achieved robust genome editing after SRI in mice (RPE-restricted editing with some Müller transduction), and certain surface chemistries unexpectedly enabled photoreceptor transfection, highlighting tunable tropism by formulation choice (113). Peptide guided LNPs have likewise expanded neural retina mRNA delivery in mice, while separate studies show mouse and human retinal tissues can be transfected with LNP-mRNA without marked inflammation, supporting translational potential (106, 114). In parallel, compacted DNA nanoparticles (CK30-PEG) demonstrated high level photoreceptor and RPE expression in mice and favorable ocular safety *in vivo*, offering a non-viral plasmid platform with large cargo capacity (115).

Another active area is precise regulation of transgene expression. Doxycycline inducible Cre in RPE and tamoxifen-inducible CreERT2 lines targeting RGCs or RPE constitute robust conditional platforms in the mouse retina, enabling spatiotemporal regulation while underscoring practical issues, including tamoxifen dosing and toxicity (116–118). Optogenetic control provides a complementary route. A clinical proof of concept in degenerative blindness demonstrated partial vision recovery with an intravitreal vector combined with light stimulating goggles, and concordant studies in NHPs using the same regimen provided supporting evidence (119).

The application of single-cell sequencing technologies is enabling deeper insights into the retinal immune microenvironment (120, 121). This approach allows researchers to better understand the complex interactions between retinal cells and the immune system, identifying potential therapeutic targets and informing the design of more effective treatments. Finally, AI-driven design is beginning to optimize vector components to improve targeting and, by enabling lower and more selective dosing, may help mitigate immune risks. Recent Machine Learning (ML) models trained on mouse *in vivo* and human *in vitro* screens accurately predict macaque AAV capsid performance, evidencing cross-species generalization, and reviews describe their integration into retinal gene delivery pipelines (122, 123).

### 5.2 Emerging strategies for immune tolerance

Cell-based Tregs therapies, encompassing polyclonal and antigen-specific Tregs and exploratory CAR-Tregs, have shown feasibility and early safety in transplantation and autoimmunity, yet remain preclinical or at best early phase for controlling AAV immunogenicity (124, 125). For retinal translation, several priorities stand out. First, generate lineage-stable FOXP3^+^ Tregs with a demethylated TSDR that are resistant to pro-inflammatory cytokine signaling (126). Second, confer specificity for the AAV capsid and the therapeutic transgene to limit bystander suppression (125). Third, achieve ocular tropism and residence, choose the optimal route (systemic, periocular, intravitreal, or subretinal), and set dose levels with a primary emphasis on safety. Finally, incorporate safety circuits such as suicide switches, and adopt standardized immune and ocular monitoring, including binding and NAbs assays, T cell readouts, inflammation grading, and optical coherence tomography (OCT) or ERG, to link immunomodulation with clinical benefit. Given the added manufacturing and clinical complexity, cell-based Treg therapy is best positioned as a mid to long-term strategy pending AAV retina specific evidence of efficacy and durability.

As efforts to translate Treg therapies continue, attention is also turning to Mesenchymal stromal cells (MSCs) derived modalities that may broaden the immunomodulatory toolkit for retinal gene therapy. MSCs and their extracellular vesicles (EVs) attenuate Th1/Th17 responses, expand Tregs, and confer neuroprotection in ocular inflammatory and degenerative models, largely via paracrine mediators such as IL-10, TGF-β, and PGE_2_ (127–129). EVs and exosomes recapitulate much of this immunomodulatory and neuroprotective activity in experimental autoimmune uveitis and retinal injury models (129, 130). Therefore, direct evidence that MSCs mitigate anti-AAV ocular immunity remains limited.

### 5.3 Translational challenges and regulatory considerations

During treatment, regular immune monitoring is essential, including the use of flow cytometry to analyze changes in T cell subsets in peripheral blood (131). Researchers have also optimized IFN-γ and IL-2-based ELISpot assays to assess T cell responses to AAV peptide antigens. Additionally, a chemiluminescence based ELISA has been developed to measure antibody levels against AAV8 in human serum, offering an alternative to traditional NAb assays. This method, owing to its simplicity and operational ease, is a valuable tool for pre-dose baseline screening (132).

**Figure 5:**
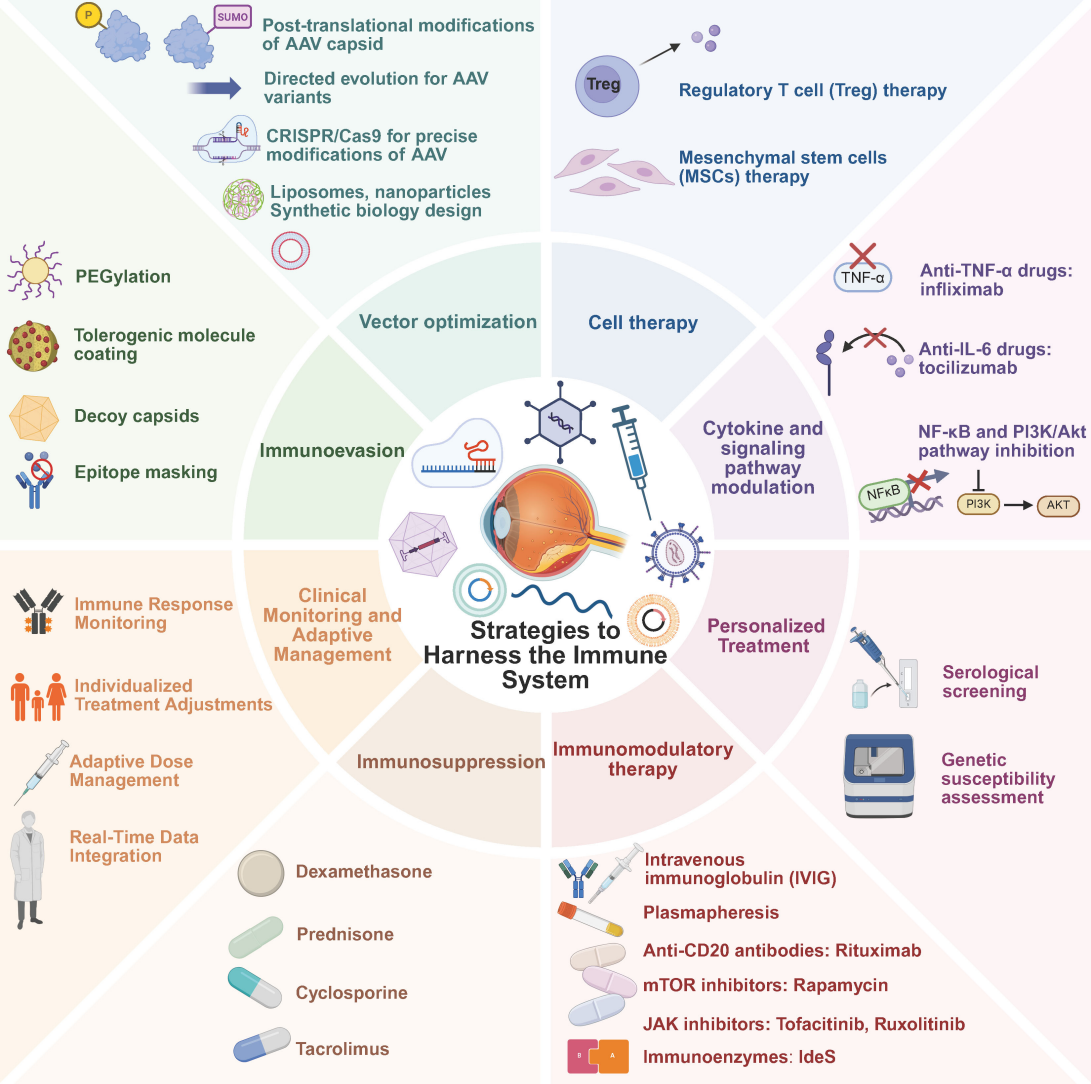
Strategies to harness immune responses in retinal gene therapy. Various strategies are currently employed to prevent and mitigate immune responses in retinal gene therapy. Personalized Treatment includes (1) serological screening and (2) genetic susceptibility assessment. Immunomodulatory Therapy encompasses (1) IVIG, (2) plasmapheresis, (3) anti-CD20 antibodies such as Rituximab, (4) mTOR inhibitors like Rapamycin, (5) JAK inhibitors such as Tofacitinib and Ruxolitinib, and (6) immunoenzymes like IdeS. Immunosuppression involves (1) dexamethasone, (2) prednisone, (3) cyclosporine, and (4) tacrolimus. Clinical Monitoring and Adaptive Management include (1) immune response monitoring, (2) individualized treatment adjustments, (3) adaptive dose management, and (4) real-time data integration. Immunoevasion strategies involve (1) PEGylation, (2) tolerogenic molecule coating, (3) decoy capsids, and (4) epitope masking. Vector Optimization includes (1) post-translational modifications of AAV capsid, (2) directed evolution for AAV variants, (3) CRISPR/Cas9 for precise modifications of AAV, (4) liposomes and nanoparticles, and (5) synthetic biology design. Cell Therapy consists of (1) Treg therapy and (2) MSCs therapy. Cytokine and Signaling Pathway Modulation involves (1) anti-TNF-α drugs such as Infliximab, (2) anti-IL-6 drugs like Tocilizumab, and (3) NF-κB and PI3K/Akt pathway inhibition.

Moreover, all clinical trials must adhere to the regulatory guidelines of the respective country, such as FDA regulations in the United States or EMA standards in Europe. Trial designs and operations must also undergo review by ethics committees to ensure compliance with ethical standards and protect participants’ rights. Any adverse events related to immune responses must be thoroughly documented and reported to allow for timely adjustments to treatment protocols, thereby improving future research. To mitigate immune risks, researchers have suggested strategies such as adjusting the dose and frequency of gene therapy administration to reduce the immune system’s burden (133, 134). **Figure 5** illustrates strategies to address immune responses in retinal gene therapy.

## 6 Conclusion

Retinal gene therapy has made substantial progress in treating IRDs. However, immune responses remain a significant challenge, impacting the safety and long-term efficacy of these therapies. Despite the retina’s immune-privileged status, AAV vectors can trigger immune reactions, leading to inflammation, uveitis, or other adverse effects. While some immune responses are inevitable, strategies such as immunosuppressive drugs, immune modulation, and vector engineering have shown promise in managing these responses. Furthermore, personalized treatment approaches, immune monitoring, and adjunctive therapies like cell therapy hold significant potential for enhancing treatment success. Future research should focus on optimizing vector design, refining immunomodulatory therapies, and improving immune monitoring techniques to ensure the safety and long-term effectiveness of retinal gene therapies. Collaboration between clinicians, immunologists, and gene therapy researchers will be essential in overcoming the immune-related barriers and realizing the full potential of gene therapy for retinal diseases.
